# Loss of Deacetylation Activity of Hdac6 Affects Emotional Behavior in Mice

**DOI:** 10.1371/journal.pone.0030924

**Published:** 2012-02-06

**Authors:** Masahide Fukada, Atsuko Hanai, Atsuo Nakayama, Takayoshi Suzuki, Naoki Miyata, Ramona M. Rodriguiz, William C. Wetsel, Tso-Pang Yao, Yoshiharu Kawaguchi

**Affiliations:** 1 Department of Embryology, Institute for Developmental Research, Aichi Human Service Center, Kasugai, Aichi, Japan; 2 Graduate School of Pharmaceutical Sciences, Nagoya City University, Nagoya, Aichi, Japan; 3 Mouse Behavioral and Neuroendocrine Analysis Core Facility, Duke University Medical Center, Durham, North Carolina, United States of America; 4 Pharmacology and Cancer Biology, Duke University Medical Center, Durham, North Carolina, United States of America; Chiba University Center for Forensic Mental Health, Japan

## Abstract

Acetylation is mediated by acetyltransferases and deacetylases, and occurs not only on histones but also on diverse proteins. Although histone acetylation in chromatin structure and transcription has been well studied, the biological roles of non-histone acetylation remain elusive. Histone deacetylase 6 (Hdac6), a member of the histone deacetylase (HDAC) family, is a unique deacetylase that localizes to cytoplasm and functions in many cellular events by deacetylating non-histone proteins including α-tubulin, Hsp90, and cortactin. Since robust expression of Hdac6 is observed in brain, it would be expected that Hdac6-mediated reversible acetylation plays essential roles in CNS. Here we demonstrate the crucial roles of Hdac6 deacetylase activity in the expression of emotional behavior in mice. We found that *Hdac6*-deficient mice exhibit hyperactivity, less anxiety, and antidepressant-like behavior in behavioral tests. Moreover, administration of Hdac6-specific inhibitor replicated antidepressant-like behavior in mice. In good agreement with behavioral phenotypes of *Hdac6-*deficient mice, Hdac6 dominantly localizes to the dorsal and median raphe nuclei, which are involved in emotional behaviors. These findings suggest that HDAC6-mediated reversible acetylation might contribute to maintain proper neuronal activity in serotonergic neurons, and also provide a new therapeutic target for depression.

## Introduction

Acetylation of the ε-amino group of lysine is a reversible post-translational modification mediated by acetyltransferases and deacetylases. This type of acetylation is not restricted to histones but also occurs on diverse proteins and affects functions such as DNA binding, protein-protein interaction, enzymatic activity, and stability. Therefore, lysine acetylation emerges as an important post-translational modification that regulates a wide range of cellular processes (reviewed in [1]).

Histone deacetylases (HDACs) are a family of enzymes with 18 isoforms in mammals, and are grouped into four classes by sequence homology [2]. HDAC6 belongs to class II, and has a unique structure with two catalytic domains and a C-terminal BUZ domain that binds ubiquitin. HDAC6 gene is located on X chromosome both in mice and human genome [3], [4]. In mice, Hdac6 protein is broadly expressed in multiple tissues, particularly abundant in the brain and testis (Fig. S1) [5]. HDAC6 is known to be a multi-functional cytoplasmic deacetylase that controls cell motility [6]–[9], endocytosis [10], vesicle transport [11], glucocorticoid receptor maturation [12], autophagic protein degradation [13]–[15], and aggresome formation [16] by deacetylating α-tubulin, cortactin, and Hsp90. These cellular events are closely related to the acquisition and maintenance of proper function in neurons. For example, at synapses, vesicle transport and endocytosis are underlying mechanisms for neurotransmitter release and recycling. Glucocorticoid receptor maturation is necessary for the negative feedback regulation of stress at the hippocampus [17]. Autophagic protein degradation and aggresome formation are a part of the quality control of proteins, and their disturbance leads to neurodegenerative disorders [18]. Despite the fact that Hdac6 protein is abundantly expressed in mice brain, the physiological implications of Hdac6 as well as non-histone acetylation in neural function are poorly understood. In this study, we found that Hdac6 is highly expressed in serotonergic neuron, and that loss of Hdac6 deacetylase activity leads to hyperactivity, less-anxiety and antidepressant-like behavior in mice. Our findings suggest that Hdac6-mediated non-histone deacetylation plays crucial roles in the expression of emotional behaviors.

## Results

### Hdac6 is localized to serotonergic neurons in raphe nuclei

**Figure 1:**
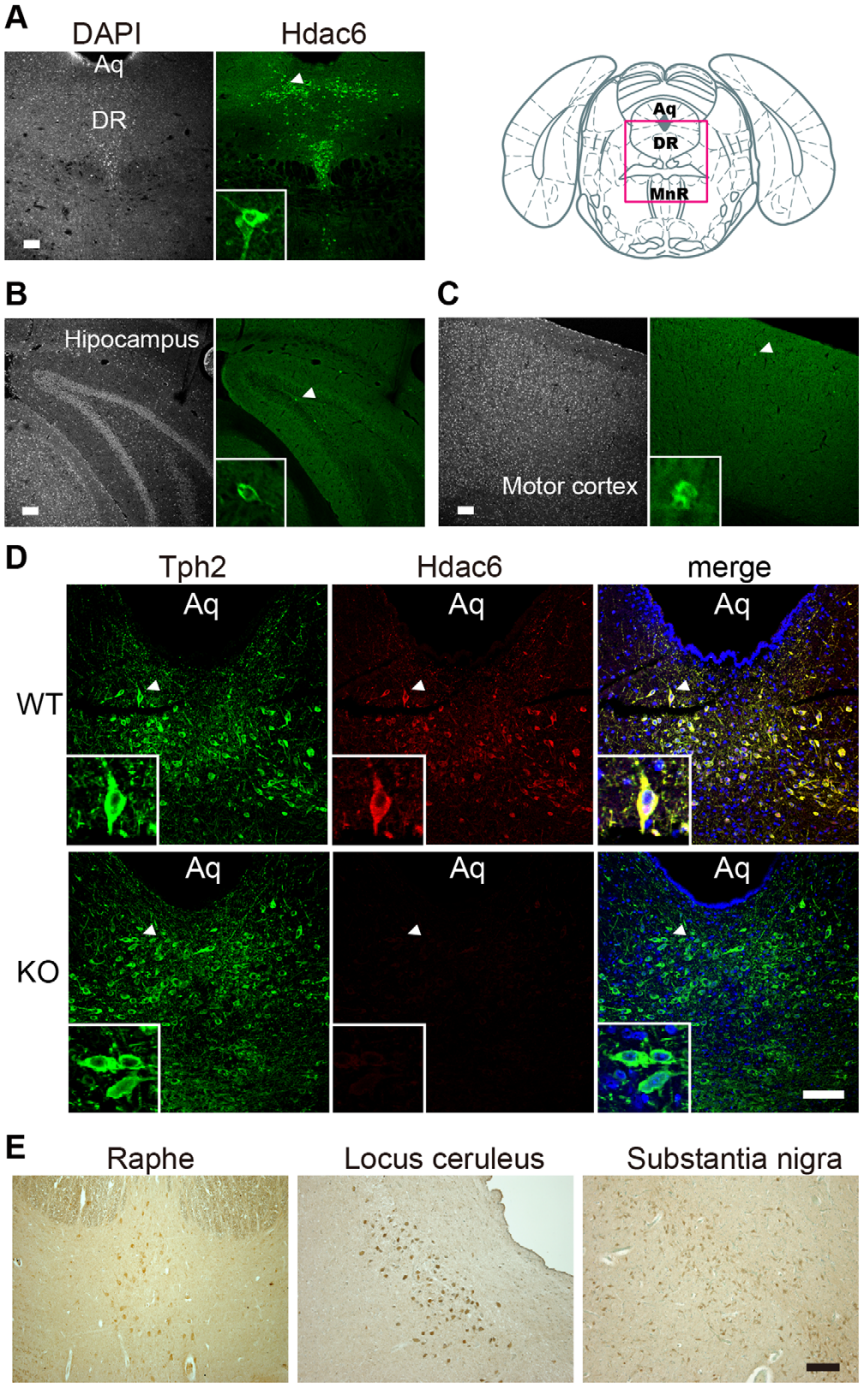
Strong expression of Hdac6 in raphe nuclei. (**A–C**) Hdac6 distribution in mouse brain. Hdac6 protein is visualized by an antibody to Hdac6 (green: each right panel) in the coronal sections of adult mouse brain spanning raphe nuclei (**A**), hippocampus (**B**), and motor cortex (**C**). Nucleus is visualized by DAPI staining (each left panel). Arrowheads indicate Hdac6-positive cells magnified in insets. (**D**) Coexpression of Hdac6 with Tph2 in dorsal raphe neuron. The coronal brain sections of the dorsal raphe from WT (upper panels) and *Hdac6* KO mice (lower panels) were double immunostained for Hdac6 (red) and Tph2 (green), a marker of mature serotonin neuron. No signal was detected with anti-Hdac6 in *Hdac6* KO mice. Merged images were shown in right panels (blue: DAPI staining). (**E**) HDAC6 expression in human brainstem. HDAC6 is visualized by antibody to HDAC6 in the horizontal sections of the postmortem human brainstem spanning raphe nuclei, locus ceruleus, and substantia nigra. DR, dorsal raphe nucleus; MnR, median raphe nucleus; Aq, aqueduct. Scale bars, 100 µm.

To study the physiological implications of Hdac6 in CNS, we first examined the distribution of Hdac6 in mouse brain by using specific antibody for mouse Hdac6. We found robust expression of Hdac6 in the medial and dorsal raphe nuclei (Fig. 1A), while Hdac6 was expressed only weakly and sparsely in hippocampus (Fig. 1B), cerebral cortex (Fig. 1C), and other brain regions (data not shown). In the medial and dorsal raphe nuclei of the WT brain, Hdac6 signal was almost exclusively co-localized with tryptophan hydroxylase 2 (Tph2) that was used as a marker for serotonin neuron (Fig. 1D) [19]. No signal was detected with Hdac6 antibody in where Tph2 signal was positive in raphe nuclei of *Hdac6* KO mice (Fig. 1D), confirming the specificity of our antibody. These results clearly indicated that mature serotonergic neurons highly express Hdac6 in mice. Similar distribution of HDAC6 was observed in human postmortem brain with additional expression of HDAC6 in substantia nigra and locus ceruleus (Fig. 1E).

The ascending serotonergic projections are derived largely from raphe nuclei and the serotonergic system contributes to regulate emotional behaviors. Indeed, the central serotonergic system is considered to be closely associated with the pathogenesis of psychiatric diseases such as depression, schizophrenia, or some anxiety disorders [20]. Therefore, these results raise the possibility that HDAC6 is involved in the expression of emotional behaviors.

### *Hdac6*-deficient mice show emotional abnormalities

**Figure 2:**
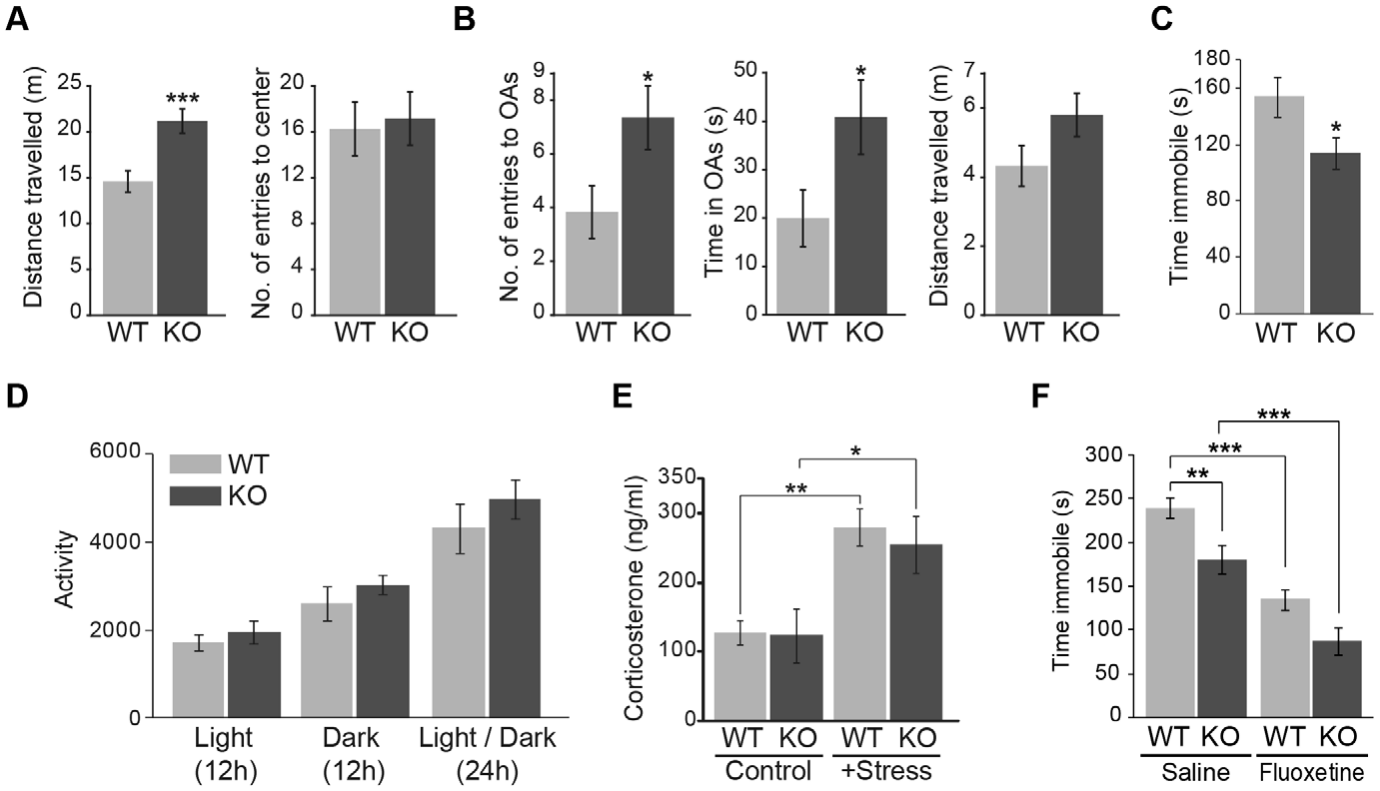
Abnormal emotional behaviors in *Hdac6* KO mice. (**A**) Hyperactivity of *Hdac6* KO mice in the open field test. Total distance traveled (left), an index of activity, and the number of entries into the center zone (right), an index of anxiety, are indicated (*n* = 24 and 23 for WT and *Hdac6* KO mice, respectively). (**B**) Anxiolytic-like behavior of *Hdac6* KO mice in the elevated plus-maze test. *Hdac6* KO mice showed increased number of entries into the open arms (OAs, left) and spent more time in OAs (middle) compared with WT mice, but total distance traveled, an index of activity, was not significantly different between genotypes (right; *n* = 24 and 23 for WT and KO, respectively). (**C**) Antidepressant-like behavior of *Hdac6* KO mice in the tail suspension test. *Hdac6* KO mice showed decreased immobility compared with WT mice (*n* = 12 for WT and KO mice). (**D**) Normal home cage activity of *Hdac6* KO mice. Home cage activity of KO mice in both light and dark periods was not distinguishable from that of WT mice (*n* = 5 and 6 for WT and KO, respectively). (**E**) Normal stress response of *Hdac6* KO mice. No genotype differences were found in serum corticosterone levels in both basal and stressful conditions (*n* = 9, 10, 10, and 10 for control WT, control KO, +stress WT, and +stress KO, respectively). (**F**) Effect of fluoxetine on the immobility of WT and *Hdac6* KO mice in the tail suspension test (*n* = 18, 18, 12, and 13 for saline WT, saline KO, fluoxetine WT, and fluoxetine KO, respectively).

*Hdac6*-deficient (KO) mice are viable and fertile, and develop normally, regardless of the hyperacetylation of α-tubulin in most tissues [5]. Our histological examination revealed no obvious difference between wild-type (WT) and *Hdac6* KO mouse brains (data not shown). To determine whether *Hdac6* KO mice suffer from emotional abnormality, we performed a series of behavioral tests. In the open field test, total distance traveled, an index of activity, was significantly elevated in *Hdac6* KO mice compared with that in WT mice (145% on average; *p*<0.001), while there were no significant differences between WT and KO mice in the number of entries into the center zone, which is an index of anxiety (Fig. 2A). These data suggest that *Hdac6* KO mice suffer from hyperactivity under novel environment. Next, we conducted an elevated plus-maze test, a commonly used test for assessing anxiety. Mice normally avoid the open arms (OAs) of the elevated plus-maze owing to anxiety. In this test, *Hdac6* KO mice showed increased number of entries into the OAs (192% on average; *p*<0.05) and spent more time in OAs compared with WT mice (205%; *p*<0.05), but total distance traveled, an index of activity, was not significantly different between genotypes (*p* = 0.094) (Fig. 2B). These results suggest that *Hdac6* KO mice have less anxiety.

To further examine the emotional aspects of *Hdac6* KO mice, we employed the tail suspension test. This is a widely used test for assessing antidepressant activity by measuring the duration of immobility during a 6 min session; administration of antidepressant decreases the immobility in rodents [21]. In this test, immobility time of *Hdac6* KO mice significantly decreased in 75% of WT mice (*p*<0.05) (Fig. 2C), indicating that *Hdac6* KO mice show antidepressant-like activity.

Behavioral abnormalities observed in *Hdac6* KO mice here seem to be a consequence of emotional arousal. However, there is still some room for argument about the possibility that behavioral abnormalities in *Hdac6* KO mice are merely due to increased locomotor activity, which is a key component of many behavioral tests. In order to assess whether basal locomotor activity was affected in *Hdac6* KO mice, we recorded the home cage activity of WT and *Hdac6* KO mice using an ANIMEX activity meter (ANIMEX, AB Farad) under a 12-h light/dark cycle for 24 h. Home cage activity of *Hdac6* KO mice in both light and dark periods, however, was not distinguishable from that of WT mice (Fig. 2D) as well as circadian rhythms (data not shown). In addition, no significant difference between WT and *Hdac6* KO mice was observed in neurophysiological screening (Table S5.). These results indicate that the basal locomotor activity and the neurophysiological functions of *Hdac6* KO mice are normal. It should be noted that the hyperactivity is only induced when *Hdac6* KO mice is exposed to novel environment. This result implies that *Hdac6* KO mice exhibit mental abnormality during unfamiliar environment.

Since stress is considered to be one of the important contributors in the etiology of depression, we investigated whether stress state and/or stress response is affected in *Hdac6* KO mice. The hypothalamic-pituitary-adrenal (HPA) axis is a principal effector of the stress response, and is often activated during depression owing to impaired negative feedback regulation, which leads to an increase in serum glucocorticoid [22]. Therefore, we determined the concentration of serum glucocorticoid (corticosterone in mice) in both basal and stressful conditions induced by the tail suspension test. However, we found no difference between WT and *Hdac6* KO mice (Fig. 2E). This suggests that the basal activity of HPA axis as well as stress response is normal in *Hdac6* KO mice. Altogether, we concluded that behavioral abnormalities observed in *Hdac6* KO mice arise from emotional arousal.

To obtain insight into the mechanism of emotional abnormalities of *Hdac6* KO mice, we focused on the alteration in the content of serotonin, an important factor of etiology of depression [20]. Since Hdac6 is concentrated in serotonergic neurons in the dorsal and median raphe nuclei, Hdac6 depletion in these neurons would affect serotonin biosynthesis, resulting in change in the content of serotonin. To examine this, we measured the amount of serotonin and Tph2, a rate-limiting enzyme of serotonin synthesis in both WT and *Hdac6* KO mice. For measuring serotonin concentration by immunoassay, we used the whole brain extract, because serotonergic neurons project to almost all regions of the brain. For evaluation of the expression level of Tph2, we used raphe nuclei extract. However, we found no obvious difference in both serotonin concentration and Tph2 level between WT and *Hdac6* KO mice (Fig. S2A and S2B). Moreover, the Tph2 distribution in the raphe nuclei of *Hdac6* KO mice was also not distinguished from that of WT mice (Fig. 1D). These results suggest that serotonin synthesis and content in *Hdac6* KO mice is normal.

Since acute administration of antidepressants reduces the immobility in the tail suspension test in mice typically by blocking serotonin reuptake and enhancing serotonin neurotransmission [20], it would be expected that *Hdac6* KO mice have already been affected in this process. In an attempt to clarify this issue, we examined the sensitivity to antidepressant in *Hdac6* KO mice. We injected fluoxetine, a selective serotonin reuptake inhibitor (SSRI), into *Hdac6* KO mice, and evaluated its potency by the tail suspension test. As a result, fluoxetine significantly decreased the immobility of *Hdac6* KO mice (48% vs. saline control; *F*_(1,57)_ = 46.2; *p*<0.0001), and its potency for *Hdac6* KO mice was similar levels to that for WT mice (56% vs. saline control) (Fig. 2F). Desipramine, a blocker of noradrenalin reuptake, and imipramine, a serotonin and noradrenalin reuptake inhibitor (SNRI), showed similar effects in *Hdac6* KO mice (Fig. S3). These results suggest that loss of Hdac6 might not affect the monoamine reuptake targeted by SSRI/SNRI.

### Administration of HDAC6 specific inhibitor leads to antidepressant-like behavior in mice

**Figure 3:**
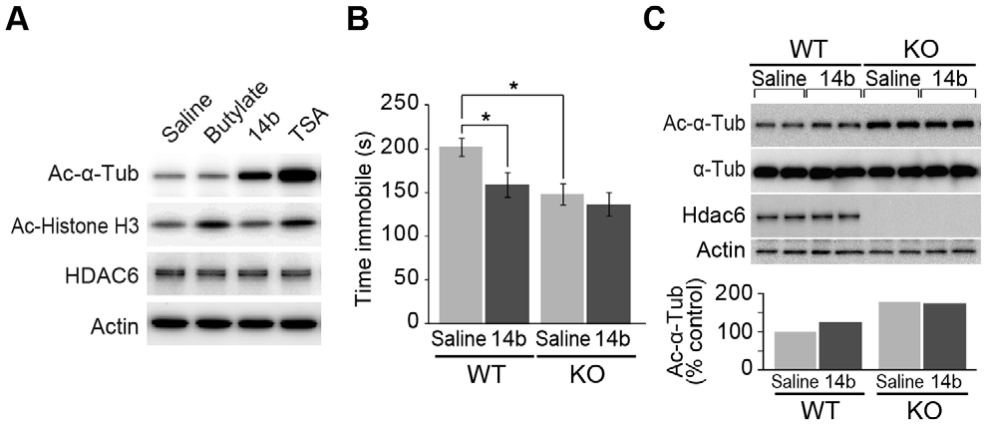
Inhibition of Hdac6 deacetylase activity causes antidepressant-like effect. (**A**) A NCT-14b (14b) treatment of the HeLa cells increased the amount of acetylated-α-tubulin (Ac-α-Tub) but not that of histone H3. HDAC6 and actin were shown as loading controls. (**B**) Effect of NCT-14b on the immobility of WT and *Hdac6* KO mice in the tail suspension test (*n* = 14, 21, 16, and 16 for saline WT, saline KO, NCT-14b WT, and NCT-14b KO, respectively). (**C**) Effect of NCT-14b on α-tubulin acetylation in mouse brain. Amount of acetylated α-tubulin in the brain extract after the tail suspension test in (**B**) was determined by Western blotting. Lower panel shows quantification of Ac-α-Tub normalized by actin.

To explore whether antidepressant-like behavior in *Hdac6* KO mice is simply due to loss of Hdac6 deacetylase activity, we adopted an approach to inhibit Hdac6 deacetylase activity in WT mice. For this purpose, we first tested the specificity of NCT-14b, one of a series of thiolate analogues that inhibit HDAC6 [23]. Since HDAC6 is localized exclusively in the cytoplasm and deacetylates α-tubulin [6], [7], [24], inhibition of Hdac6 activity would increase the amount of acetylated α-tubulin, but not that of acetylated histones. As expected, NCT-14b treatment of the HeLa cells increased the acetylation of α-tubulin but not that of histone H3, demonstrating the specificity of NCT-14b for HDAC6 (Fig. 3A). In contrast, sodium butyrate, a well-known class I HDAC inhibitor that does not inhibit HDAC6, only increased the acetylation of histone H3, while a non-specific potent HDAC inhibitor, trichostatin A (TSA), increased the acetylation of both α-tubulin and histone H3 (Fig. 3A).

In the tail suspension test, intraperitoneal administration of NCT-14b significantly reduced the immobility of WT mice (79% vs. saline-injected control; *F*_(1,63)_ = 4.22; *p* = 0.044) (Fig. 3B). In contrast, the same amount of NCT-14b had little effect on the immobility of *Hdac6* KO mice, indicating that NCT-14b targets Hdac6 to reduce immobility. It should be noted that the immobility-reducing effect of NCT-14b reached at almost equal to that of *Hdac6* gene knockout (75% vs. WT mice; *p*<0.05; see Fig. 2C). This indicates that the antidepressant-like behavior of *Hdac6* KO mice is not only due to the long-term effect of gene depletion, but also caused by the transient loss of Hdac6 deacetylase activity in the adult brain. To evaluate the inhibition potency of NCT-14b *in vivo*, brains of two mice from each group in Figure 3B were isolated, and then the acetylation level of α-tubulin was examined. As expected, NCT-14b treatment modestly increased the amount of acetylated α-tubulin in WT mice brain (Fig. 3C). To examine the local effects in the raphe nuclei of NCT-14b administration on α-tubulin acetylation , we injected same concentration of NCT-14b into WT mice, and evaluated the levels of α-tubulin acetylation in isolated raphe nuclei specimen. As shown in Figure S4, modest increase of α-tubulin acetylation was observed in raphe nuclei of NCT-14b-injected mice. These results confirm the validity of NCT-14b in *in vivo* application, and demonstrate that inhibition of the deacetylase activity of Hdac6 causes antidepressant-like behavior in mice.

## Discussion

In the present study, we provided evidence that non-histone protein acetylation plays an important role in CNS by focusing on Hdac6, a multifunctional cytoplasmic deacetylase. We demonstrated that disturbance of Hdac6-mediated protein deacetylation both by gene knockout and by chemical inhibition of deacetylase activity results in behavioral consequences related to emotion in mice. Emotional features such as fear and depression are known to be closely associated with the serotonergic system. In good agreement with this, Hdac6 is most abundant in serotonergic neurons in the dorsal and median raphe nuclei, the origin of the ascending serotonergic fibers innervating the forebrain and amygdala [25], [26]. Thus, it is plausible that Hdac6-mediated reversible acetylation in serotonergic neurons regulates its neuronal functions.

Recently, an antidepressant-like effect of HDAC inbitors, N-(2-aminophenyl)-4-[N- (pyridine-3-ylmethoxy-carbonyl) aminomethyl]benzamide (MS-275; classI HDACs inhibitor) and suberoylanilide hydroxamic acid (SAHA; class I and II HDACs inhibitor), in social defeat paradigms has been reported in mice [27]. Moreover, Hdac2 deficiency as well as chronic treatment with SAHA in mice leads to memory facilitation by increasing the synapse number in hippocampus CA1 region [28]. In these reports, transcriptional regulation controlled by the dynamic change of histone acetylation either in the nucleus accumbens [27] or hippocampus [28] is responsible for antidepressant-like effect or memory facilitation, respectively. In contrast, since the level of histones acetylation is not affected by both *Hdac6* gene knockout (Fig. S2B) [24] and chemical inhibition of HDAC6 activity in Hela cells (Fig. 3A), it is clear that abnormality of emotional behavior caused by Hdac6 inhibition does not result from alteration of transcriptional regulation. To our knowledge, this is the first report to identify the association between non-histone protein acetylation and higher brain function.

The elevated plusmaze test and the tail suspension test, adopted in this study, are well-established behavioral experiments assessing the anxiety and depression, respectively. Although *Hdac6* KO mice exhibited less anxiety and antidepressant-like activity in these tests, we should point out the possibility that such behavior is, in part, responsible for increased locomotor activity under a novel environment. In this regard, we consider that such novelty-induced abnormal hyperactivity represents emotional problems.

Although, at present, the molecular mechanism of how inhibition of Hdac6-mediated protein deacetylation leads to hyperactivity, less-anxiety, and antidepressant-like activity still remains elusive, we have obtained some insight into this issue. First, the mechanism underlying antidepressant-like behavior observed in both *Hdac6* KO mice and NCT-14b-injected WT mice might be different from that of current antidepressants, which target serotonin and noradrenalin transporters and enhance the neurotransmission [20]. Our results showing that the antidepressants are still effective in *Hdac6* KO mice in the tail suspension test (Fig. 2F) and serotonin content is not affected in *Hdac6* KO mice (Fig. S2A) support this view. Second, Hdac6 may contribute to regulate emotional behavior by deacetylating other Hdac6 substrate(s) besides α-tubulin. It should be noted that the amount of acetylated α-tubulin in NCT-14b-injected WT mice brain as well as dorsal raphe region does not reach the same level as that of *Hdac6* KO mice (Figs. 3C, S4) even though NCT-14b fully affects the immobility in WT mice (Fig. 3B). Thus, there is a discrepancy between acetylation levels of α-tubulin and immobility in NCT-14b-injected mice, and it raises the possibility that Hdac6 has a specific substrate other than α-tubulin in serotonergic neurons. Further study is needed to find a specific target for Hdac6, which would be a key for serotonergic function associated with an antidepressant-like behavioral response.

Our findings also suggest that HDAC6-mediated reversible acetylation can become a new therapeutic target for depression. Since NCT-14b seems to act as a non-monoamine-based antidepressant, HDAC6 inhibitors may have potential as medicines to overcome the disadvantage of current antidepressants, namely, their requirement of at least several weeks for therapeutic action [20]. Identification of HDAC6 substrates and related cellular processes in serotonergic neurons associated with the expression of antidepressant-like behavior will bring new insights into the molecular basis of mood disorders.

## Materials and Methods

### Animals

*Hdac6* KO mice [10] were backcrossed at least six times with 129SV background. Because Hdac6 gene is located on X chromosome [3], male mice carrying the mutant allele are null for Hdac6. Mice were maintained on a 12-h light/dark cycle (lights on at 7:00 AM) and were allowed access to food and water *ad libitum*. We used male mice at 3–12 months of age for all experiments. All experimental procedures in this study and housing condition were reviewed and approved by the animal experimentation committee of the Institute for Developmental Research in Aichi Human Service Center (Permit number: M-19).

### Antibodies

Rabbit polyclonal antibody against mouse Hdac6 was as described previously [10]. Anti-acetylated lysine monoclonal antibody (clone AKL5C1) was generously provided by Dr. Minoru Yoshida (RIKEN, Saitama, Japan). Anti-histone H3 rat monoclonal antibody was generous gift from Dr. Hiroshi Kimura (Osaka University, Osaka, Japan) [29]. Commercially available antibodies for α-tubulin (Cedarlane), Lys40-acetylated α-tubulin (Sigma), human HDAC6 (C300, Santa Cruz Biotechnology), acetylated histone H3 (Millipore), tryptophan hydroxylase 2 (NB100-74555, Novus biologicals), and pan-actin (NeoMarkers) were also used in this study. For the double immunostaining procedure, rabbit polyclonal antibody against mouse Hdac6 was directly coupled with the Alexa Fluor 555 dye using the Alexa Fluor 555 Monoclonal Antibody Labeling Kit (Invitorgen) according to the manufacturer's instructions.

### Chemicals

Fluoxetine hydrochloride, desipramine hydrochloride, and imipramine hydrochloride were purchased from Sigma. These drugs were dissolved in saline and injected intraperitoneally in a volume of 10 ml/kg body weight 30 min prior to testing. Drug doses were 25 mg/kg imipramine, 30 mg/kg fluoxetine, and 20 mg/kg desipramine. These effective doses were chosen on the basis of previous reports on mice [21]. Trichostatin A (TSA) and sodium butyrate were purchased from Sigma. An HDAC6-selective inhibitor, the compound (S)-S-7-(adamant-1-ylamino)-6-(tert-butoxycarbonyl)-7-oxoheptyl-2-methylpropanethioate (NCT-14b) [23], dissolved in saline at 4.8 mg/kg, was injected intraperitoneally in a volume of 10 ml/kg body weight 24 h prior to testing. We compared the effects of NCT-14b treatment period either 30 min or 24 h by the tail suspension test and found that 24 h treatment more effectively reduced immobility of WT mice (our unpublished data).

### Behavioral procedures

All behavioral procedures were undertaken during the light period under 100 lux light intensity. Before behavioral testing, mice were placed in an experimental room for 30 min prior to testing. For the open field test, a mouse was placed in the center of an open field apparatus (white wooden box: 40×40×20 cm, divided into 9 even-sized squares (13×13 cm)), and recorded using a video camera positioned above the apparatus. The total distance traveled in the open field was recorded for 10 min to measure the horizontal activity. The number of entries into the square at the center of the field was counted to evaluate anxiety. For the elevated plus-maze test, a mouse was placed into the center of the plus-maze apparatus (Ohara-Ika Sangyo), which was raised 50 cm above the floor, consisting of two open arms (40 cm long by 10 cm wide) and two enclosed arms of the same size with walls 20 cm high, and recorded using a video camera positioned above the apparatus. The number of entries into the open arms was counted over a 10 min test session to evaluate anxiety. A video-tracking system with computer interface and video camera (Any-maze; Stoelting) was used to automatically collect behavioral data. In this system, the animal's entries into defined areas of the apparatus were counted when 80% of animal's entire area was onto respective zone. For the tail suspension test, a mouse was fastened by the distal end of the tail to a plastic clip (Yamashita-Giken) and was suspended in a white wooden box (40×25×25 cm). The presence of immobility, defined as the absence of any movement, was counted over a 6 min test session by a highly trained observer who remained blind to genotype and treatment. For the analysis of home cage activity, home cage with a mouse was placed on an ANIMEX activity meter (AB Farad) and mouse activity was measured for 24 h.

### Immunohistochemistry

For immunohistochemistry, mice were anesthetized and transcardially perfused with phosphate-buffered saline (PBS) followed by 4% paraformaldehyde in PBS. Brains were removed and cryostat sections (10 µm thick, coronal sections) were prepared with standard protocols. For double immunostaining for Hdac6 and TPH2, the brain section was firstly reacted with anti-TPH2 antibody (1∶200 dilution), followed by Cy2-conjugated secondary antibody, and then fixed by 3% paraformaldehyde in PBS. Next, the section was reacted with the Alexa Fluor 555 labeled anti-mouse Hdac6 antibody and 4′,6′-diamidino-2-phenylindole (DAPI) to visualize Hdac6 and the nuclei, respectively. The immunofluorescent signals were observed under confocal laser scanning microscopy (FLUOVIEW FV1000; Olympus). For the detection of human HDAC6 in postmortem human brain, paraffin sections (5 µm thick, coronal sections) were prepared with standard protocols. Sections spanning raphe and locus ceruleus were derived from adult human brain, and sections spanning substantia nigra were from a newborn infant brain. Paraffin sections were stained with anti-human HDAC6 antibody (1∶200 dilution) followed by HRP-labeled secondary antibody, and were observed by light microscopy (BX61; Olympus). All experimental procedure using postmortem human brain have been reviewed and approved by the Human Ethics Committee of the Institute for Developmental Research in Aichi Human Service Center (approved number: 03-02).

### Biochemical analysis

To confirm the specificity of NCT-14b, HeLa cells (purchased from ATCC) were treated with NCT-14b (1 µM), TSA (0.1 µM), or sodium butyrate (5 mM) for 1 h. Then, cells were lysed in Laemmli sample buffer and subjected to Western blotting with antibodies against acetylated α-tubulin (1∶2000 dilution), acetylated histone H3 (1∶1000 dilution), human HDAC6 (1∶1000 dilution), and actin (1∶2000 dilution). For the quantification of acetylated α-tubulin in the brain, each mouse was killed by cervical dislocation, and brain was quickly removed and homogenized in 5 ml of buffer (5 mM Tris-HCl pH7.6, 0.32 M sucrose, 1 mM EDTA, 1 µM TSA) with a glass-teflon homogenizer. The homogenates were centrifuged at 1000 g for 10 min to remove nuclei and debris. The supernatants (brain extract) were then subjected to Western blotting with an ECL detection system (GE Healthcare) as described previously [30]. The dorsal raphe-containing slice (2 mm thick) of mouse brain was cut out with razor blades directed by a chilled precision brain slicer (BS-2000C, Braintree Scientific, Inc.), and the dorsal raphe region were trimmed with a 1 mm biopsy punch (BP-10F, Kai Industries Co. Ltd) and processed for Western blotting. Serum corticosterone concentration was determined by immunoassay according to the manufacturer's instructions (AssayMax Corticosterone ELISA Kit, Assaypro). To evaluate the stress response, serum was prepared 30 min after the end of the tail suspension test, and was subjected to immunoassay. The serotonin concentration of both serum and brain extract was measured by immunoassay according to the manufacturer's instructions (Serotonin ELISA kit, GenWay Biotech).

### Statistical analysis

Student's *t* test was used for statistical comparison (Figs. 2A–C, S1A). For multiple comparisons, groups were compared using two-way analysis of variance, followed by Bonferroni's post hoc test (Figs. 2E, 2F, 3B and S3). The data are presented as mean ± s.e.m., *n* indicates the sample number, and *p* denotes the significance (*p<0.05, **p<0.01, ***p<0.001).

## Supporting Information

Figure S1 **Abundant expression of Hdac6 in the brain and testis in mice.** Two micrograms of tissue extract was analyzed by Western blotting with anti-Hdac6 antibody.
(TIF)

Figure S2 **Normal serotonin content in** ***Hdac6*** **KO mice.** (**A**) Serotonin concentration of both brain extracts and serum of *Hdac6* KO mice was determined by immunoassay. Serotonin concentrations of *Hdac6* KO mice were comparable to that of WT mice (brain extracts; WT, 18.8±1.2 ng/mg protein (*n* = 5), *Hdac6* KO mice, 20.4±2.3 ng/mg protein (*n* = 6), serum; WT, 1154 ng±43 ng/mg protein (*n* = 3), *Hdac6* KO mice, 1274±252 ng/mg protein (*n* = 4). (**B**) The expression levels of indicated proteins in dorsal raphe region of WT and *Hdac6* KO mice brain were analyzed by Western blotting. Acetylation level of α-tubulin in *Hdac6* KO mice was higher than that of WT mice. In contrast, the acetylation of histone H3 in *Hdac6* KO mice was similar to WT mice. The Tph2, a rate limiting enzyme of serotonin synthesis in the brain, was abundant in dorsal raphe compared to cortex, and the amount of Tph2 in *Hdac6* KO mice was comparable to that of WT mice. The amount of α-tubulin, histone H3 and actin were shown as loading controls.
(TIF)

Figure S3 **Effects of imipramine and desipramine on** ***Hdac6*** **KO mice in the tail suspension test.** Effects of acute injection of imipramine (25 mg/kg) and desipramine (20 mg/kg) on the immobility of WT and *Hdac6* KO mice in the tail suspension test was investigated. Imipramine significantly reduced both immobility times of WT (56% on average) and *Hdac6* KO mice (59%) compared with each of saline-injected control mice (*n* = 18, 19, 18, and 16 for saline WT, saline KO, imipramine WT, and imipramine KO, respectively; *F*_(1,67)_ = 39.47; *p*<0.0001). Desipramine showed similar results as reducing immobity of WT (62%) and *Hdac6* KO mice (60%) (*n* = 18, 18, 11, and 12 for saline WT, saline KO, desipramine WT, and desipramine KO, respectively; *F*_(1,55)_ = 76.52; *p*<0.0001). Data were presented as mean ± s.e.m., and statistically analyzed by two-way analysis of variance followed by Bonferroni's post hoc test.
(TIF)

Figure S4 **Effects of NCT-14b administration on tubulin acetylation in dorsal raphe.** Amounts of acetylated α-tubulin (Ac-α-Tub) in cortex and dorsal raphe 24 h after NCT-14b administration were analyzed by Western blotting. Lower panel showed quantification of Ac-α-Tub normalized by α-tubulin (α-Tub). Although NCT-14b (14b) slightly increased the amount of Ac-α-Tub in dorsal raphe, it did not reach the same level as that of *Hdac6* KO mice (KO). The effect of NCT-14b was more pronounced in motor cortex than in dorsal raphe.
(TIF)

Table S1 **Neurophysiological screen in WT and** ***Hdac6*** **KO mice.**
(TIF)
