# Supplementary figures and images for: Loss of Deacetylation Activity of Hdac6 Affects Emotional Behavior in Mice
Source: PLoS One. 2012 Feb 6;7(2):e30924. doi: 10.1371/journal.pone.0030924 (PMC3273475; doi:10.1371/journal.pone.0030924)

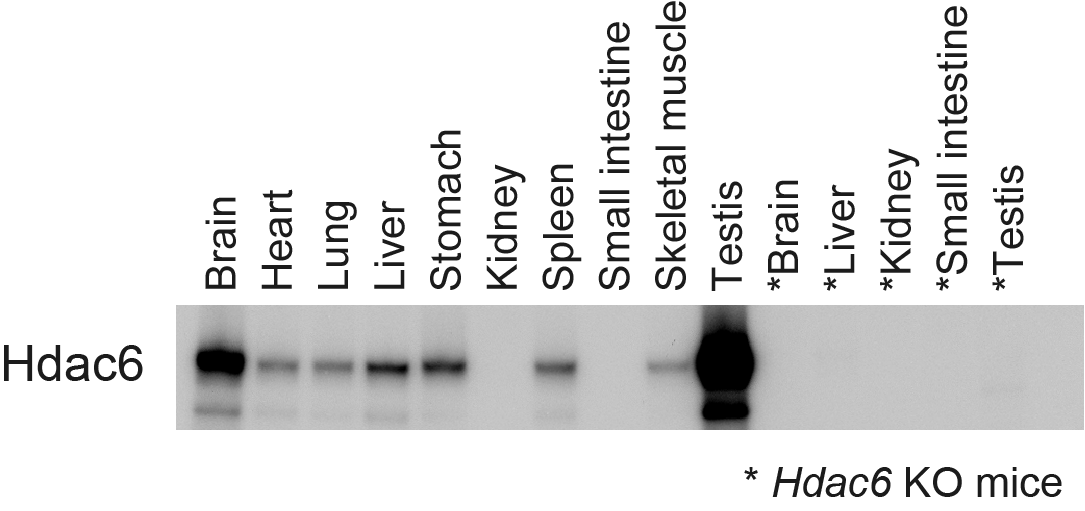

Supplement: Figure S1 — Abundant expression of Hdac6 in the brain and testis in mice. Two micrograms of tissue extract was analyzed by Western blotting with anti-Hdac6 antibody. (TIF) [file pone.0030924.s001.tif]

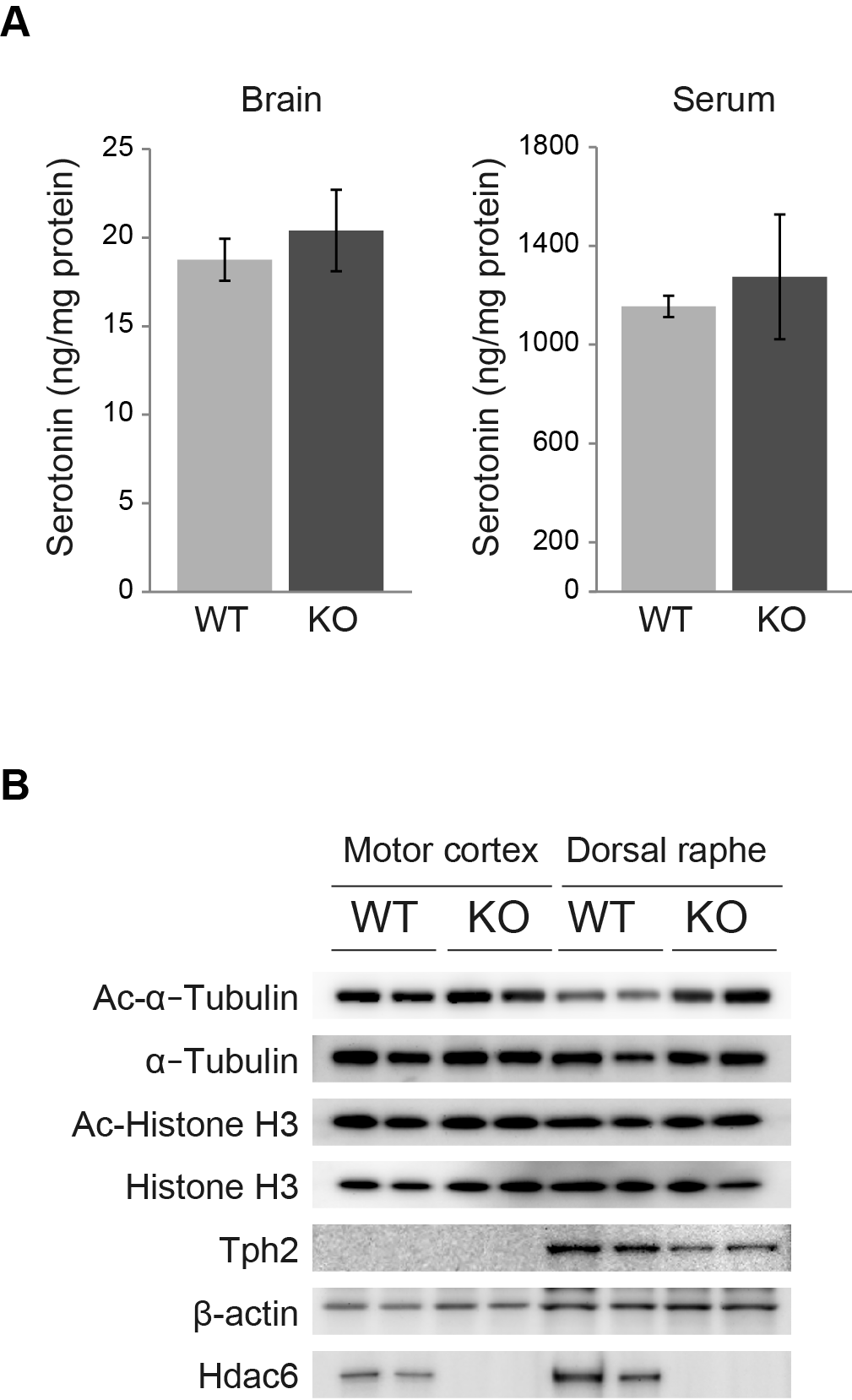

Supplement: Figure S2 — Normal serotonin content in Hdac6 KO mice. (A) Serotonin concentration of both brain extracts and serum of Hdac6 KO mice was determined by immunoassay. Serotonin concentrations of Hdac6 KO mice were comparable to that of WT mice (brain extracts; WT, 18.8±1.2 ng/mg protein (n = 5), Hdac6 KO mice, 20.4±2.3 ng/mg protein (n = 6), serum; WT, 1154 ng±43 ng/mg protein (n = 3), Hdac6 KO mice, 1274±252 ng/mg protein (n = 4). (B) The expression levels of indicated proteins in dorsal raphe region of WT and Hdac6 KO mice brain were analyzed by Western blotting. Acetylation level of α-tubulin in Hdac6 KO mice was higher than that of WT mice. In contrast, the acetylation of histone H3 in Hdac6 KO mice was similar to WT mice. The Tph2, a rate limiting enzyme of serotonin synthesis in the brain, was abundant in dorsal raphe compared to cortex, and the amount of Tph2 in Hdac6 KO mice was comparable to that of WT mice. The amount of α-tubulin, histone H3 and actin were shown as loading controls. (TIF) [file pone.0030924.s002.tif]

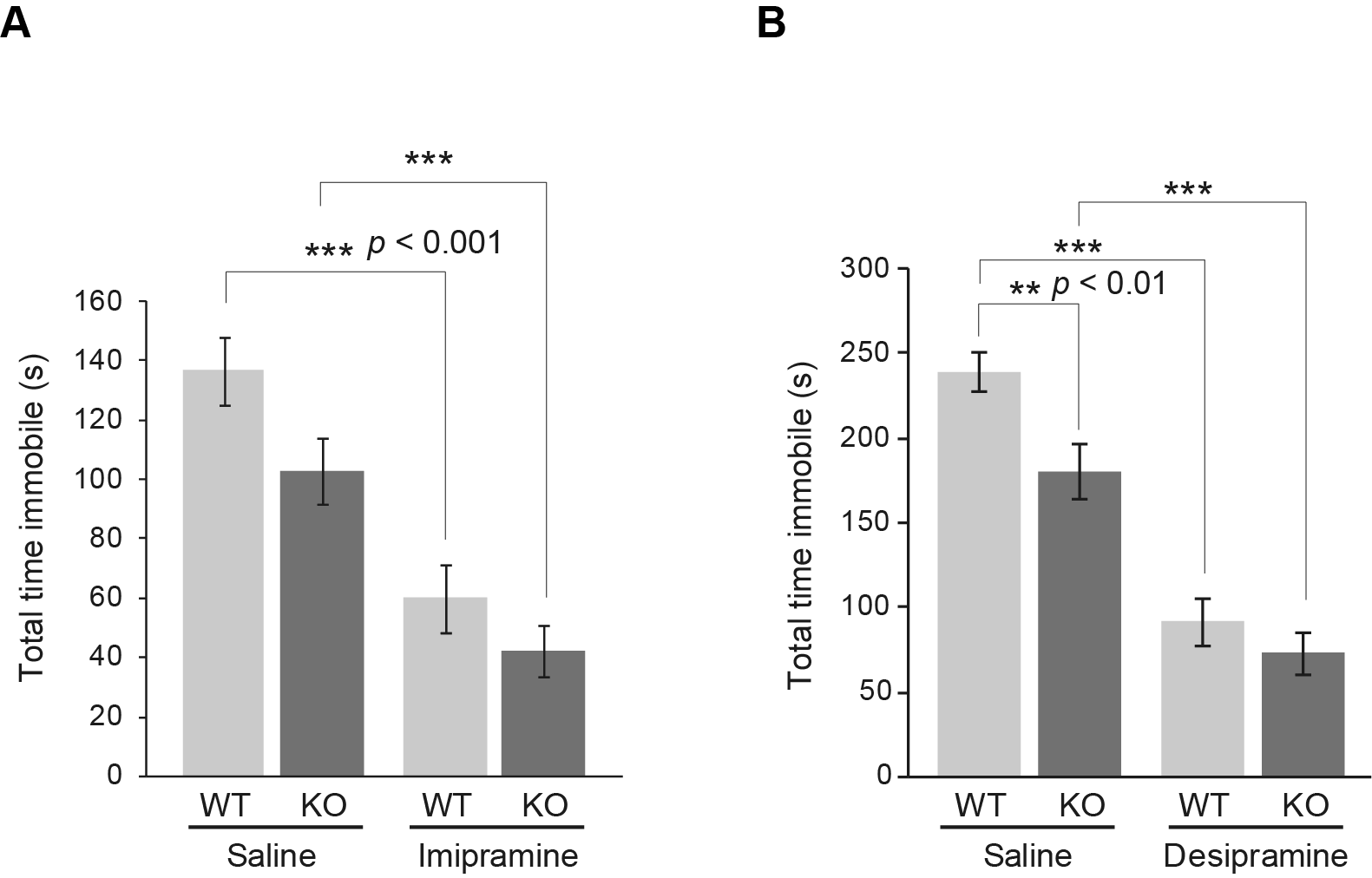

Supplement: Figure S3 — Effects of imipramine and desipramine on Hdac6 KO mice in the tail suspension test. Effects of acute injection of imipramine (25 mg/kg) and desipramine (20 mg/kg) on the immobility of WT and Hdac6 KO mice in the tail suspension test was investigated. Imipramine significantly reduced both immobility times of WT (56% on average) and Hdac6 KO mice (59%) compared with each of saline-injected control mice (n = 18, 19, 18, and 16 for saline WT, saline KO, imipramine WT, and imipramine KO, respectively; F (1,67) = 39.47; p<0.0001). Desipramine showed similar results as reducing immobity of WT (62%) and Hdac6 KO mice (60%) (n = 18, 18, 11, and 12 for saline WT, saline KO, desipramine WT, and desipramine KO, respectively; F (1,55) = 76.52; p<0.0001). Data were presented as mean ± s.e.m., and statistically analyzed by two-way analysis of variance followed by Bonferroni's post hoc test. (TIF) [file pone.0030924.s003.tif]

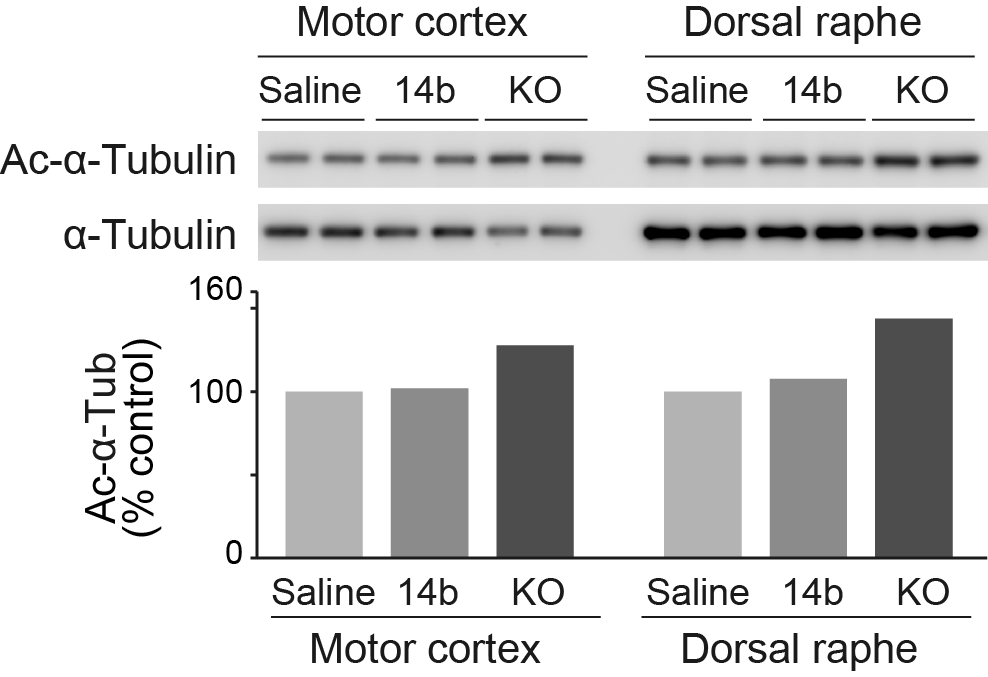

Supplement: Figure S4 — Effects of NCT-14b administration on tubulin acetylation in dorsal raphe. Amounts of acetylated α-tubulin (Ac-α-Tub) in cortex and dorsal raphe 24 h after NCT-14b administration were analyzed by Western blotting. Lower panel showed quantification of Ac-α-Tub normalized by α-tubulin (α-Tub). Although NCT-14b (14b) slightly increased the amount of Ac-α-Tub in dorsal raphe, it did not reach the same level as that of Hdac6 KO mice (KO). The effect of NCT-14b was more pronounced in motor cortex than in dorsal raphe. (TIF) [file pone.0030924.s004.tif]

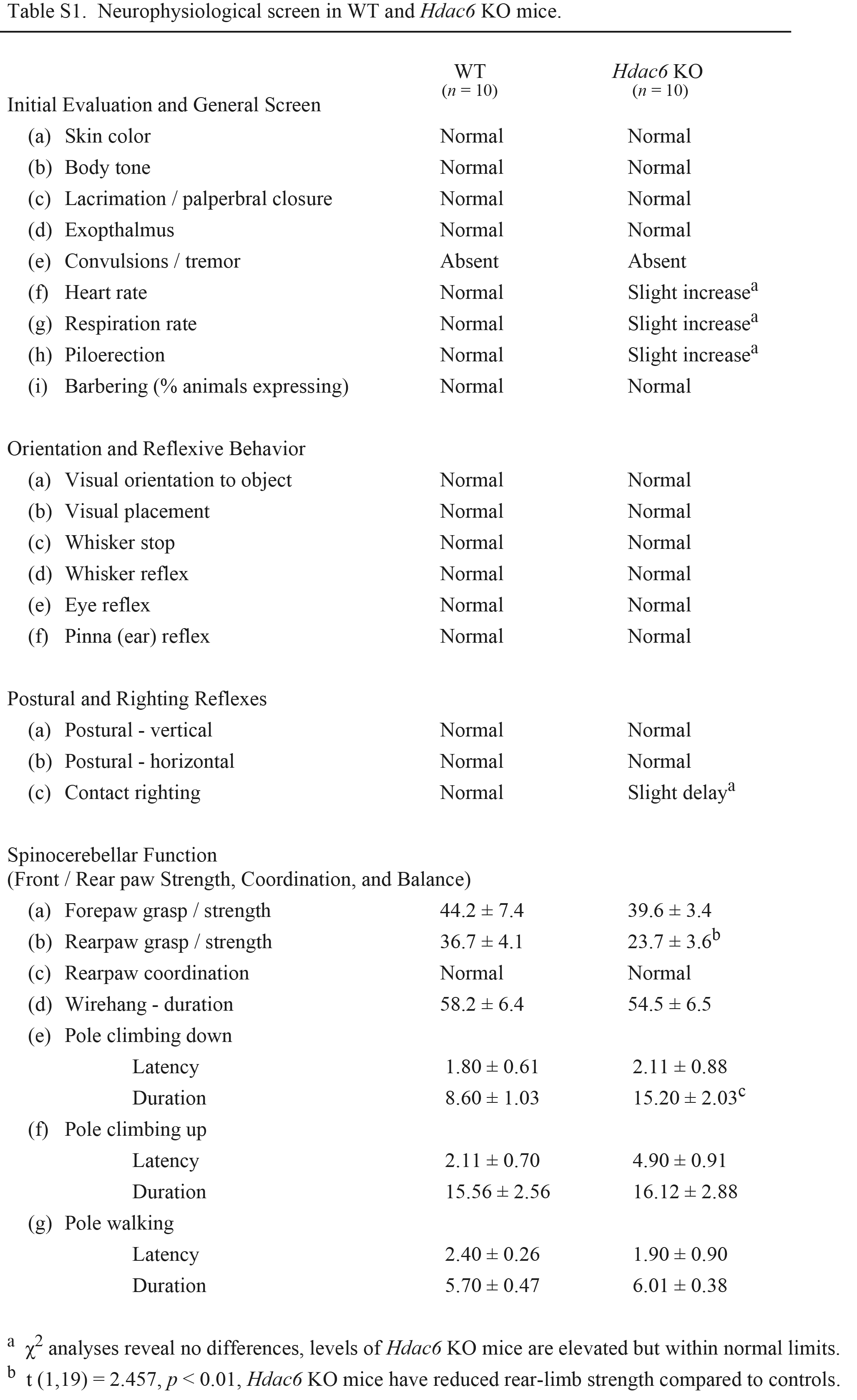

Supplement: Table S1 — Neurophysiological screen in WT and Hdac6 KO mice. (TIF) [file pone.0030924.s005.tif]
